# Supplementary material for: Exosome-Derived microRNAs from Mouthrinse Have the Potential to Be Novel Biomarkers for Sjögren Syndrome
Source: J Pers Med. 2022 Sep 10;12(9):1483. doi: 10.3390/jpm12091483 (PMC9505666; doi:10.3390/jpm12091483)
Supplement: Supplementary file 1 [file jpm-12-01483-s001.zip › Supplementary_material_JPM final.pdf]

Supplementary Material

Mouthrinse Is a Novel Non-Invasive Sampling Method for the Diagnosis and Screening of Sjögren Syndrome

Kouta Yamashiro [1], Tomofumi Hamada [1,2], Kazuki Mori [1], Keitaro Nishi [1], Maya Nakamura [1], Mahiro Beppu [1], Akihiko Tanaka [1], Hiroshi Hijioka [1], Yoshiaki Kamikawa [1] and Tsuyoshi Sugiura [1]

[1] Department of Maxillofacial Diagnostic and Surgical Science, Field of Oral and Maxillofacial Rehabilitation, Graduate School of Medical and Dental Science, Kagoshima University, Kagoshima, Japan
[2] Department of Oral & Maxillofacial Surgery, Hakuaikai Medical Cooperation, Sagara Hospital, Kagoshima, Japan

* Correspondence: sugiura@dent.kagoshima-u.ac.jp

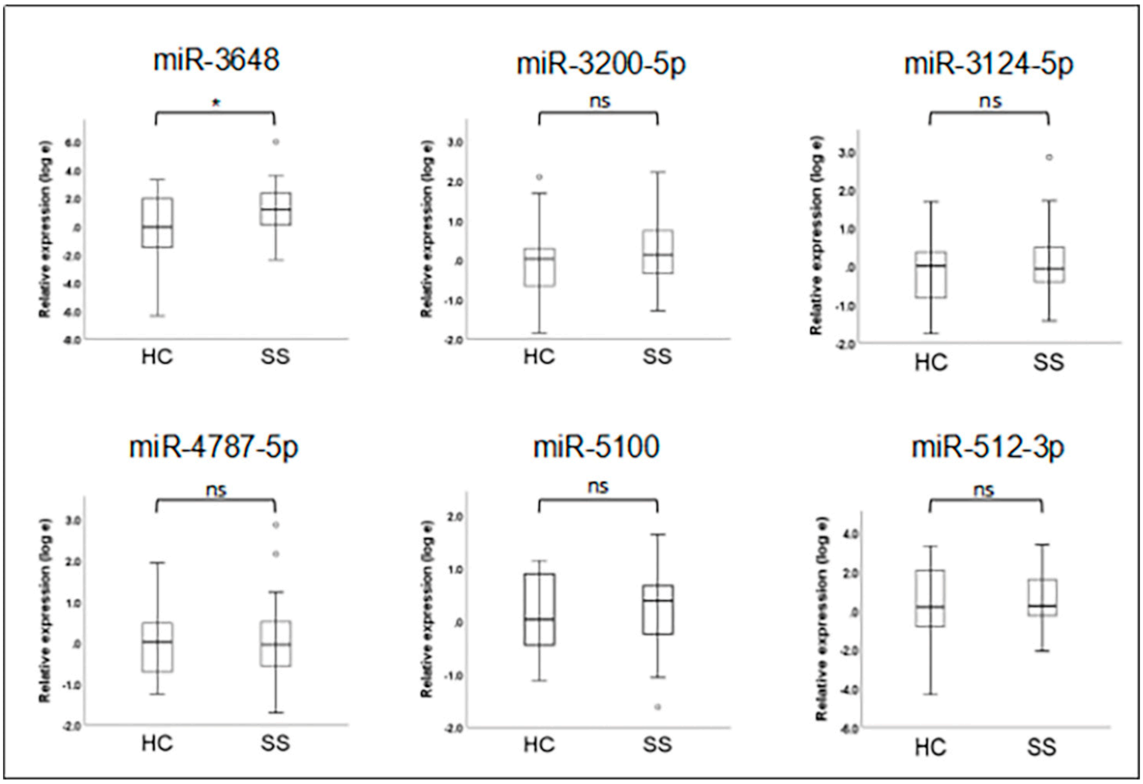

Supplementary Figure S1. Comparison of normalized signal intensity of six microRNAs between patients with Sjögren’s syndrome (SS) and controls (HC). There were significant differences in signal intensity for miR-3648 ($p < 0.05$, t tests). Signal intensity did not significantly differ for the other five miRNAs. *: $p < 0.05$, ns: no significant difference.

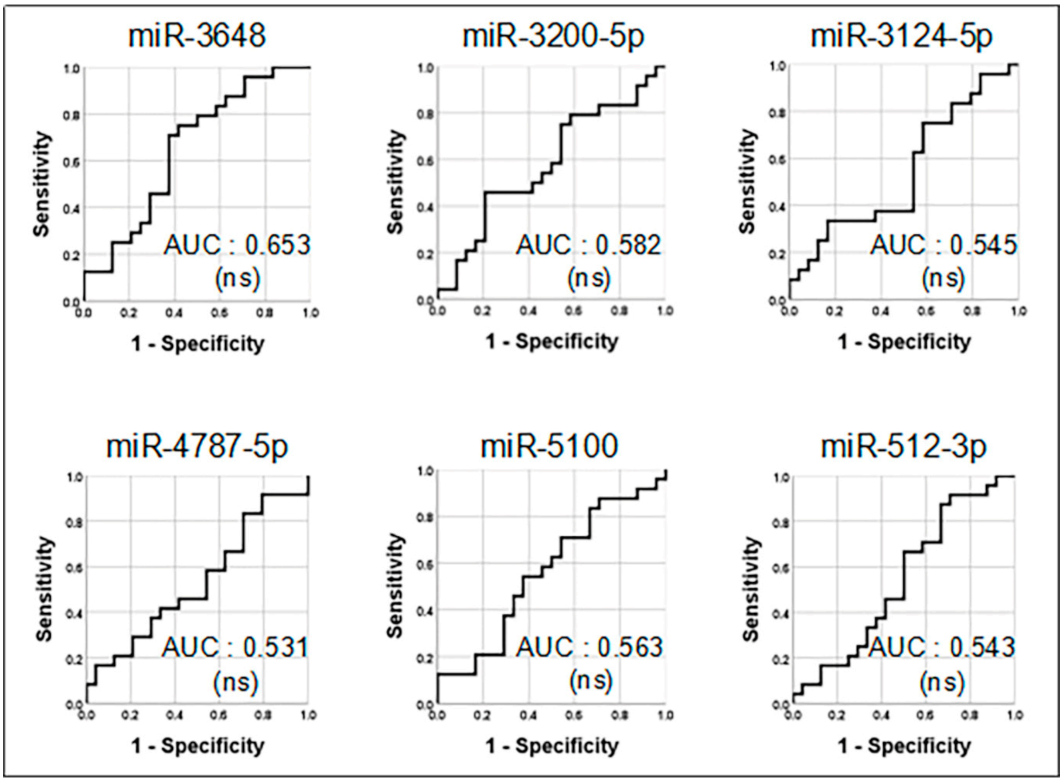

Supplementary Figure S2. AUC of six microRNAs. The six microRNA's area under ROC curves (AUC) did not significantly discriminate Sjögren's syndrome (SS) patients from controls. ns: no significant difference.
